# Lifetime activity scores in women at risk for Alzheimer's disease and relationship with midlife memory and executive function

**DOI:** 10.1002/alz70860_106055

**Published:** 2025-12-23

**Authors:** Catherine Chiao, Genna M Mashinchi, Filippo Cieri, Jessica ZK Caldwell

**Affiliations:** ^1^ Cleveland Clinic, Las Vegas, NV, USA; ^2^ Nevada Exploratory Alzheimer's Disease Research Center, Las Vegas, NV, USA

## Abstract

**Background:**

Healthy lifestyle is linked with better brain health outcomes in aging and may reduce dementia risk. The Dynamic Neurocognitive Adaptation Scale (dNA) assesses level of lifespan engagement in physical, cognitive, creative, and social activity. This analysis examined relationship of lifetime activity with current memory scores in midlife women with a family history of Alzheimer's disease. We predicted that higher dNA scores would relate to better memory performance.

**Method:**

Data were assessed for 47 women (mean age=51) who completed dNA and baseline cognitive testing. Bivariate Spearman correlation analyses examined the relationship between dNA scores (total, and sum for each domain and time window) and Rey Auditory Verbal Learning Test (RAVLT) delayed recall and Face Name Associative Memory Exam Test (FNAME) total recall scores. Exploratory analysis examined dNA relationships with Trails B, Boston Naming Test, Mini‐Mental State Examination and verbal fluency (FAS and Animals).

**Result:**

DNA subdomains were intercorrelated (rs =.51‐73, ps< .001). Highest total mean activity (M = 51.27), as well as in domains of cognitive (M = 12.16), physical (M = 10.37), and creative activities (M = 18.49) was observed in adolescence, while social activity peaked in youth (M = 10.33). Greater total dNA social domain activity was correlated with poorer RAVLT delayed recall (*r* = ‐.37, *p* = .01). There were no other significant correlations between memory scores and dNA total, domain, or time window scores. In exploratory analyses, better verbal fluency was correlated with higher total dNA (*r* = .34, *p* = .02), and higher creative (*r* = .36, *p* = .01), and physical domains (*r* = .32, *p* = .03).

**Conclusion:**

Findings demonstrate that in women at risk for Alzheimer's, engagement levels peak in adolescence and youth, and that domains of activity engagement are intercorrelated. Exploratory analysis showed positive correlations between dNA and verbal fluency, suggesting a link between cognitive flexibility and higher lifespan activity engagement. Moreover, reduced engagement in adulthood suggests women may be in need of lifestyle intervention at midlife. However, the single, negative correlation of dNA with delayed recall memory was unexpected. Lack of findings may be due to low power or may reflect our younger sample of cognitively healthy women.

